# Assembling mitogenome of Himalayan Black Bear (*U. t. laniger*) from low depth reads and its application in drawing phylogenetic inferences

**DOI:** 10.1038/s41598-020-76872-y

**Published:** 2021-01-12

**Authors:** Amrita Bit, Mukesh Thakur, Sujeet Kumar Singh, Bheem Dutt Joshi, Vinay Kumar Singh, Lalit Kumar Sharma, Basudev Tripathy, Kailash Chandra

**Affiliations:** Zoological Survey of India, New Alipore, Kolkata, West Bengal 700053 India

**Keywords:** Evolutionary biology, Genomics, Sequencing, Conservation biology, Evolutionary ecology, Molecular ecology

## Abstract

The complete mitogenome of Himalayan black bear (*Ursus thibetanus laniger*) from Indian Himalayan region was assembled following the modified approach of mitochondrial baiting and mapping using the next-generation sequencing reads. The complete mitogenome was of 16,556 bp long, consisted of 37 genes that contained 13 protein-coding genes, 22 tRNAs, 2 rRNAs and 1 control region. The complete base composition was 31.33% A, 15.24% G, 25.45%C, and 27.98%T and gene arrangement was similar to the other sub-species of Asiatic black bear. The relative synonymous codon usage analysis revealed the maximum abundance of Isoleucine, Tyrosine, Leucine and Threonine. The assembled mitogenome of *U. t. laniger* exhibited 99% similarity with the mitogenomes of Himalayan black bear available from Nepal and Tibetan Plateau-Himalaya region. The findings of the present study has proven low depth sequencing data, adequate and highly efficient in rapid recovering the mitochondrial genome by overcoming the conventional strategies of obtaining long-range PCR and subsequently drawing phylogenetic inferences.

## Introduction

The Asiatic black bear (*Ursus thibetanus*) with wide range distribution, consists of seven well recognized subspecies, *i.e.* Japanese black bear (*U*. *t*. *japonicus*) in Japan, Ussuri black bear (*U*. *t*. *ussuricus*) in far-east Russia, northeast China, and Korea, Formosan black bear (*U*. *t*. *formosanus*) in Taiwan, Indochinese/Sichuan black bear (*U*. *t*. *mupinensis*) in Southwest China, Baluchistan black bear (*U*. *t*. *gedrosianus*) in South Pakistan and Iran, Tibetan black bear (*U*. *t*. *thibetanus*) in the eastern Himalayas and southeast Asia, and Himalayan black bear (*U*. *t*. *laniger*) in the western Himalayas^1^. Among the seven sub-species of Asiatic black bear, the Himalayan black bear (henceforth, HBB) is distributed in between 1200 and 3300 m asl all along the forested habitats of the Himalayas and hills of northeastern states of India covering an area of about 270,000 km^2^ with an estimated population of 5400 to 6700 divides^2,3^. A small population of HBB is patchily distributed across Pakistan, northwest India, and likely northeast India and Nepal^4^. In India, HBB has experienced several challenges including habitat loss, population decline due to hunting/poaching for pelts, paws and gall bladders and retaliatory killing in the response to Human–Bear Conflicts^3,5,6^. Considering the increased threats and species vulnerability in wild, HBB is listed as *Vulnerable* in the Red list of IUCN^1^ and categorized under the Schedule-I of Indian Wildlife (Protection) Act 1972. Complete mitochondrial genomes of six sub-species of Asiatic black bear except the *U*. *t*. *gedrosianus* have been sequenced using long range PCR strategy^7–10^. However, no study has provided the detailed genome organization and comparative assessment for gene arrangements and structural consistency in the t-RNA model, important in variety of cellular processes controlling species life history traits.

Further, Next Generation Sequencing (NGS), which rapidly captures the broad spectrum of mutations and has dramatically revolutionized DNA sequencing^11^, and has been popularized to address questions in the field of molecular ecology^12^, phylogeographic^13^, population genetics^14^ and phylogenetic studies^15^. Most studies in bears have made use of the conventional strategy of combining long-range PCR with subsequent primer walking for sequencing the complete mitogenomes^7–10,16^. However, conventional sequencing is tedious and challenging in particular for optimizing long range PCRs. In contrast, revolution in NGS technology has made considerable decrease in cost and increase in throughput (millions of short sequencing reads) and accuracy^17,18^. Several studies have demonstrated the application of NGS in drawing the phylogenetic inferences, genome organization and comparative assessment among the sympatric species by mapping and assembly the complete mitogenomes from low depth sequencing reads^19–21^.

Therefore, to overcome the unwieldy process of conventional sanger sequencing, we assembled the complete mitogenome directly from low depth NGS reads following a modified approach of mitochondrial baiting and mapping reported earlier by Hahn et al*.*^22^. We demonstrated organization of complete mitogenome of HBB for the first time and presented its structure consistency of tRNA model with the other sub-species of Asiatic black bear. We also testified the assembled mitogenome of *U*. *t*. *laniger* in re-construction of bear phylogeny among the other black bear subspecies.

## Results and discussion

### Genome organization

**Figure 1:**
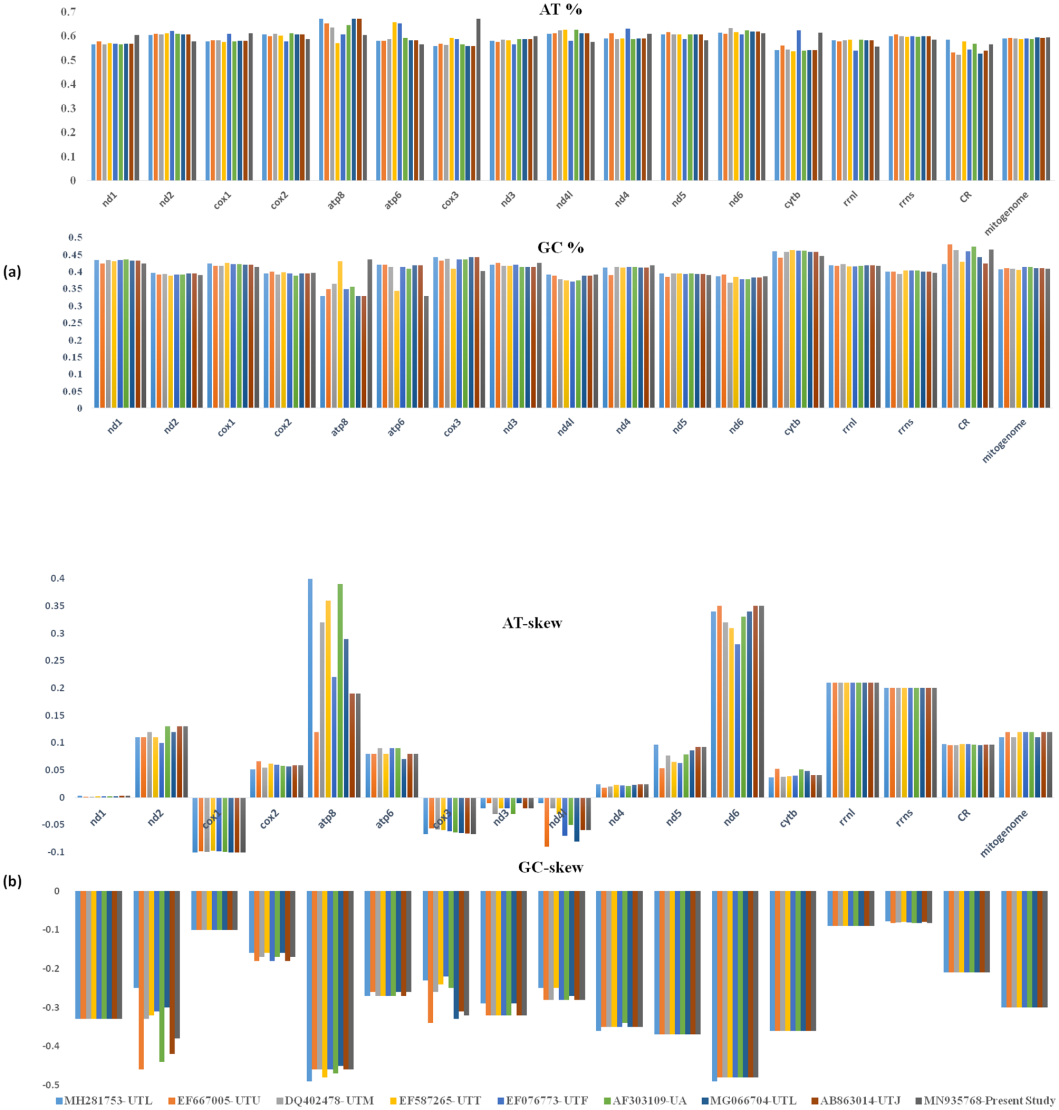
Representation of AT, GC content and skewness of Himalayan black bear with other sub-species of Asiatic black bears. (**a**) AT and GC content (**b**) and skewness.

**Figure 2:**
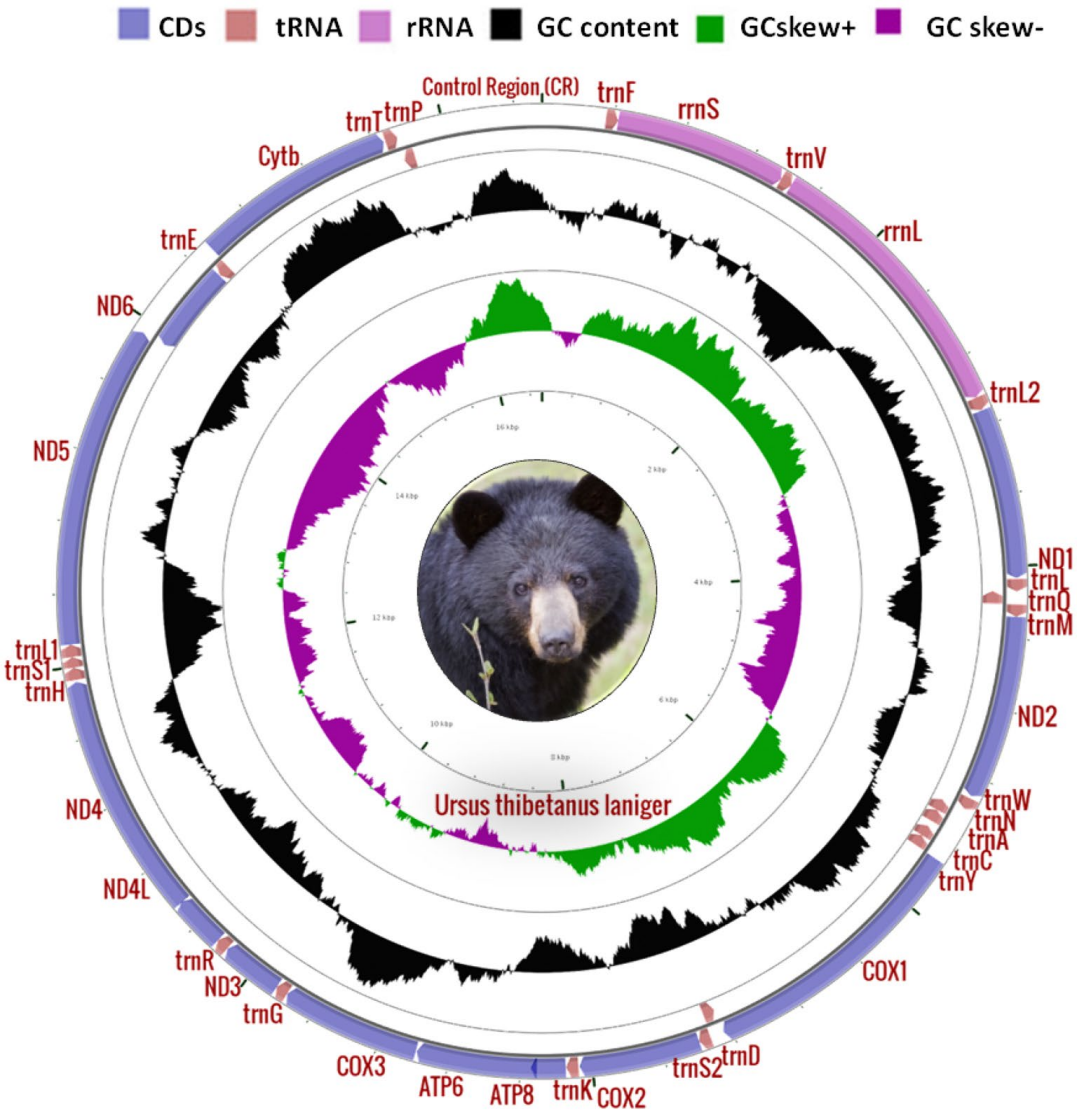
The mitochondrial genome of *Ursus thibetanus laniger.* Direction of gene signal is indicated by arrows. Protein-coding genes are shown in silver color arrows, rRNA genes in purple color arrows, tRNA genes in light pink color arrows and non-coding region in white color. The GC content is plotted using a black color; GC-skew is plotted using green and dark pink color. The figure was drawn using CGView online server (https://stothard.afns.ualberta.ca/cgview_server/) with default parameters.

A total of 3.73 GB data of ~ 1.6 × coverage was obtained from Illumina HiSeq 2500 platform which yielded 12,418,314 reads. With reference-based assembly, we obtained the longest contig of 16, 556 bp length that represented the complete mitochondrial genome of *U. t. laniger* and submitted to GenBank (accession no. MN935768). The observed total AT and GC contenst were 59.3% and 40.7% (Fig. 1a), and mitogenome showed positive AT skewness (+ 0.057), indicating that adenine bases occurred more frequently than the thymine, whereas GC-skew was negative, − 0.25. The assembled mitogenome encoded 37 genes, of which, 13 were PCGs, 22 tRNAs, 2rRNAs and one control region. The arrangement and distribution of genes were similar to the other mammalian species^23^. The overall nucleotide composition was: 31.33% -A, 25.45% -C, 15.24% -G, 27.98%- T. In exception to ND6 and eight tRNA genes (trnQ, trnA, trnN, trnC, trnY, trnS2, trnE and trnP), most genes were encoded on the heavy strand (Fig. 2). The five pairs of overlapping regions in mitogenome were observed among trnV/rrnL, trnI/trnQ, atp8/atp6, nd4l/nd4, and trnT/trnP. The overlapping regions ranged from − 1 to − 34 bp. The smallest overlapping region was located in between trnV/trnL and trnT/trnP (1 bp) whereas the longest overlapping was in between ATP8/ATP6 (40 bp). Besides, 20 intergenic spacers were observed between the mitochondrial regions ranging from 1 to 33 bp length; the longest space was found between trnN/trnC (Table 1).

**Table 1:** List of annotated mitochondrial genes in Himalayan black bear, *Ursus thibetanus laniger*.

S. No	Gene	Start	End	Size	Strand	Score	Start Codon	Intergenic Nucleotides
1	trnF(ttc)	356	423	68	+	1.05E−12		0
2	rrnS	424	1389	966	+	1.84E−61		0
3	trnV(gta)	1390	1455	66	+	8.75E−11		− 1
4	rrnL	1455	3036	1582	+	3.08E−38		1
5	trnL2(tta)	3038	3112	75	+	1.47E−07		2
6	nd1	3115	4065	951	+	2.24E+08	ATG	5
7	trnI(atc)	4071	4139	69	+	1.10E−12		− 3
8	trnQ(caa)	4137	4209	73	−	1.30E−08		1
9	trnM(atg)	4211	4279	69	+	4.13E−08		0
10	nd2	4280	5308	1029	+	1.74E+08	ATA	13
11	trnW(tga)	5322	5388	67	+	1.47E−12		8
12	trnA(gca)	5397	5465	69	−	1.40E−07		0
13	trnN(aac)	5466	5538	67	−	9.13E−10		33
14	trnC(tgc)	5572	5638	73	−	1.88E−11		0
15	trnY(tac)	5639	5705	67	−	1.66E−09		1
16	cox1	5707	7245	1539	+	2.91E+08	ATG	3
17	trnS2(tca)	7249	7317	67	−	1.36E−07		6
18	trnD(gac)	7324	7390	69	+	2.25E−08		0
19	cox2	7391	8071	681	+	1.31E+08	ATG	6
20	trnK(aaa)	8078	8145	68	+	1.13E−09		1
21	atp8	8148	8342	195	+	1226437	ATG	− 34
22	atp6	8309	8983	675	+	82486767	ATG	5
23	cox3	8989	9771	783	+	2.02E+08	ATG	1
24	trnG(gga)	9773	9841	69	+	5.61E−11		0
25	nd3	9842	10186	345	+	20858803	ATG	1
26	trnR(cga)	10189	10257	69	+	5.22E−09		0
27	nd4l	10258	10551	294	+	12258664	ATG	− 4
28	nd4	10548	11915	1368	+	2.68E+08	ATG	10
29	trnH(cac)	11926	11994	69	+	6.07E−09		0
30	trnS1(agc)	11995	12053	59	+	1.28E−06		0
31	trnL1(cta)	12054	12123	70	+	3.38E−14		0
32	nd5	12124	13929	1806	+	3.16E+08	ATG	4
33	nd6	13934	14452	519	−	27691523	TCC	3
34	trnE(gaa)	14456	14524	69	−	2.66E−09		4
35	cytb	14529	15659	1131	+	2.43E+08	ATG	9
36	trnT(aca)	15669	15738	70	+	1.93E−09		− 1
37	trnP(cca)	15738	15803	66	−	5.68E−13		0

### PCGs and rRNAs

All 13 PCGs had ATG start codon except nd2 and nd6 which encoded by ATA and TCC, respectively. The total length of PCGs was 11,316 bp which shared 68.3% of complete mitogenome (Table 2). The average base composition in PCGs were 30.1%- A, 28.6%- T, 14.2%- G and 27.2%- C. The abundance of AT (%) was higher than GC (%).Comparative analysis of *U. t. laniger* with the other subspecies of Asiatic black bear and *Ursus americanus* exhibited relatively high adenine and cytosine contents than thymine and guanine. All the PCGs showed positive AT skew except for the genes cox1, cox3, nd3 and nd4l whereas GC skew showed negative skewness for all the genes (Fig. 1b). The PCGs region consisted of twelve heavy strands and one light strand as commonly found in other vertebrate species^24–26^. The PCGs region consisted of seven NADH dehydrogenases, three cytochrome c oxidases, two ATPases and one cytochrome b genes.

**Table 2:** Nucleotide composition and skewness in the Himalayan black bear, *Ursus thibetanus laniger* mitochondrial genome.

*U.t. laniger*	Size (bp)	A%	T%	AT-skew	G%	C%	GC-skew
Whole mitogenome	16556	31.33	27.98	0.057	15.24	25.45	− 0.25
PCGs	11316	30.1	28.6	0.025	14.2	27.2	− 0.314
tRNAs	1508	32.9	31.2	0.026	19.4	16.4	0.083
rRNAs	2548	35.9	23.5	0.208	18.5	22.0	− 0.086
Control region	1109	27.3	31.4	− 0.069	17.7	23.6	− 0.142

The mtDNA ribosomal region is known to be highly conserved and widely used for phylogenies of higher and middle category level, such as phyla, family and genera^24,26^. The length of 12S rRNA and 16S rRNA genes was 966 bp and 12,582 bp, respectively. The 12S rRNA gene was positioned between the tRNA-Phe and tRNA-Val and 16S rRNA gene was positioned between tRNA-Val and tRNA-Leu2. Similar to PCGs, the AT skewness was positive (0.208) and the GC skewness was negative − 0.086) (Table 2) and the total AT content of rRNA was 59.4% which was in correspondence with other sub-species of Asiatic black bear (Table 2).

### Transfer RNAs and control region

**Figure 3:**
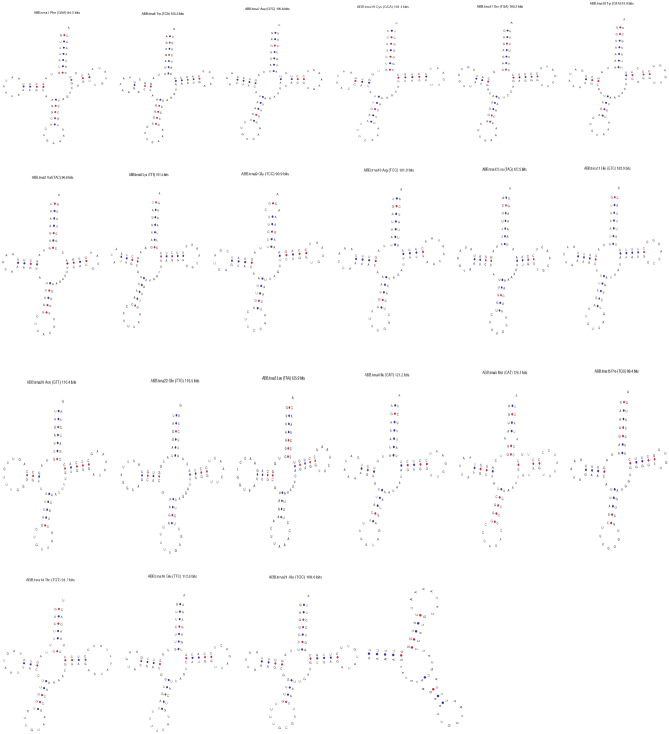
Putative secondary structures of 22 transfer RNA genes of *Ursus thibetanus linager* where the red dots indicated Watson–crick pairing and the blue dots indicated other non-Watson–Crick interactions.

The length of the tRNA was 1508 bp, overall AT and GC content was 64.1% and 35.9% respectively. The average AT and GC skewness values for tRNAs were 0.026 and 0.083, respectively (Table 3). The results exhibited 21 tRNAs can fold into cloverleaf structure except for tRNA^ser^ which lacks the dihydrouridine arm (Fig. 3). The tRNA genes length varied from 59 to 75 bp and out of 22 tRNAs, fourteen were located on heavy strand and eight were on the light strand (Table 1).

**Table 3:** Comparative assessment of nucleotide composition indices among different subspecies of Asiatic black bear and other species in the Ursidae family.

S. No	GenBank ID	Whole Mitogenome		Protein coding Genes (PCGs)		Ribosomal RNA (rRNA)		Species/subspecies (Country)	Reference/sequencing methods (Sanger/NGS)
		Size (bp)	AT (%)	Size (bp)	AT (%)	Size (bp)	AT (%)		
1	MH281753	16771	59.1	11402	59.0	2546	59	*Ursus thibetanus laniger (Nepal)*	Kadariya et al.^10^/Sanger sequencing
2	EF667005	16701	59.1	11416	59.0	2549	59	*Ursus thibetanus ussuricus* (South Korea)	Hwang et al.^9^/Sanger sequencing
3	DQ402478	16868	59.2	11410	59.0	2545	60	*Ursus thibetanus mupinensis* (China)	Hou et al.^8^/Sanger sequencing
4	EF196661	16795	59.0	11405	59.0	2541	60	*Ursus thibetanus* (China)	Li et al.^8^*/S*anger sequencing
5	EF587265	17034	58.8	11211	59.0	2548	60	*Ursus thibetanus thibetanus (Taiwan)*	Chou et al. (unpublished data)/Sanger sequencing
6	EF076773	17044	58.7	11418	59.0	2549	59	*Ursus thibetanus formosanus* (Taiwan)	Hsieh et al. 2016 (unpublished data)/Sanger sequencing
7	MG066704	16795	59.1	11405	59.0	2648	60	*Ursus thibetanus* (Tibetan Plateau)	Lan et al.^33^/Sanger sequencing
8	FM177759	16893	58.9	11403	58.0	2548	60	*Ursus thibetanus* (Germany)	Krause et al., 2008 (unpublished data)/Sanger sequencing
9	AB863014	16748	59.4	11405	59.0	2540	60	*Ursus thibetanus japonicus* (China)	Wu et al.^34^/Sanger sequencing
10	JX196366	16434	59.3	11400	59.0	2546	60	*Ursus americanus (USA)*	Miller et al. 2012/Genome sequencing
11	AF303109	16841	59.4	11409	59.0	2545	60	*Ursus americanus* (Canada)	Delisle and Strobeck^16^/Sanger sequencing
12	AF303110	17020	58.7	11409	59.0	2541	60	*Ursus arctos* (Canada)	Delisle and Strobeck^16^/Sanger sequencing
13	GU573490	16808	58.9	11406	59.0	2542	59	*Ursus maritimus* (USA)	Lindqvist et al. 2010/Sanger sequencing
14	EF196664	16783	59.0	11410	59.0	2546	60	*Helarctos malayanus* (China)	Li et al.^8^/sanger sequencing
15	NC 009970	16817	58.3	11406	58.0	2545	59	*Melursus ursinus* (China)	Li et al.^8^/Sanger sequencing
16	EF196665	16766	58.6	11410	58.0	2553	60	*Tremarctos ornatus* (China)	Li et al.^8^/Sanger sequencing
17	EF212882	16805	61.2	10957	62.0	2547	61	*Ailuropoda melanoleuca* (China)	Peng et al.*/*sanger sequencing
18	MN935768	16556	59.3	11316	59.0	2548	59	*Ursus thibetanus laniger (India; Present Study)*	Present Study/Low depth genome approach

The control region was located between trnP and trnF and the length was 1109 bp in size and contributed to 6.7% of the whole mitogenome with containing a microsatellite repeat, (AT)_4_ and seven 10 bp tandem repeats (Table S1). The A + T composition was 58.7%, higher than that of G + C content. The AT and GC skewness values were negative, − 0.069 and − 0.142, respectively (Table 2).

### RSCU and reconstruction of bear phylogeny

**Figure 4:**
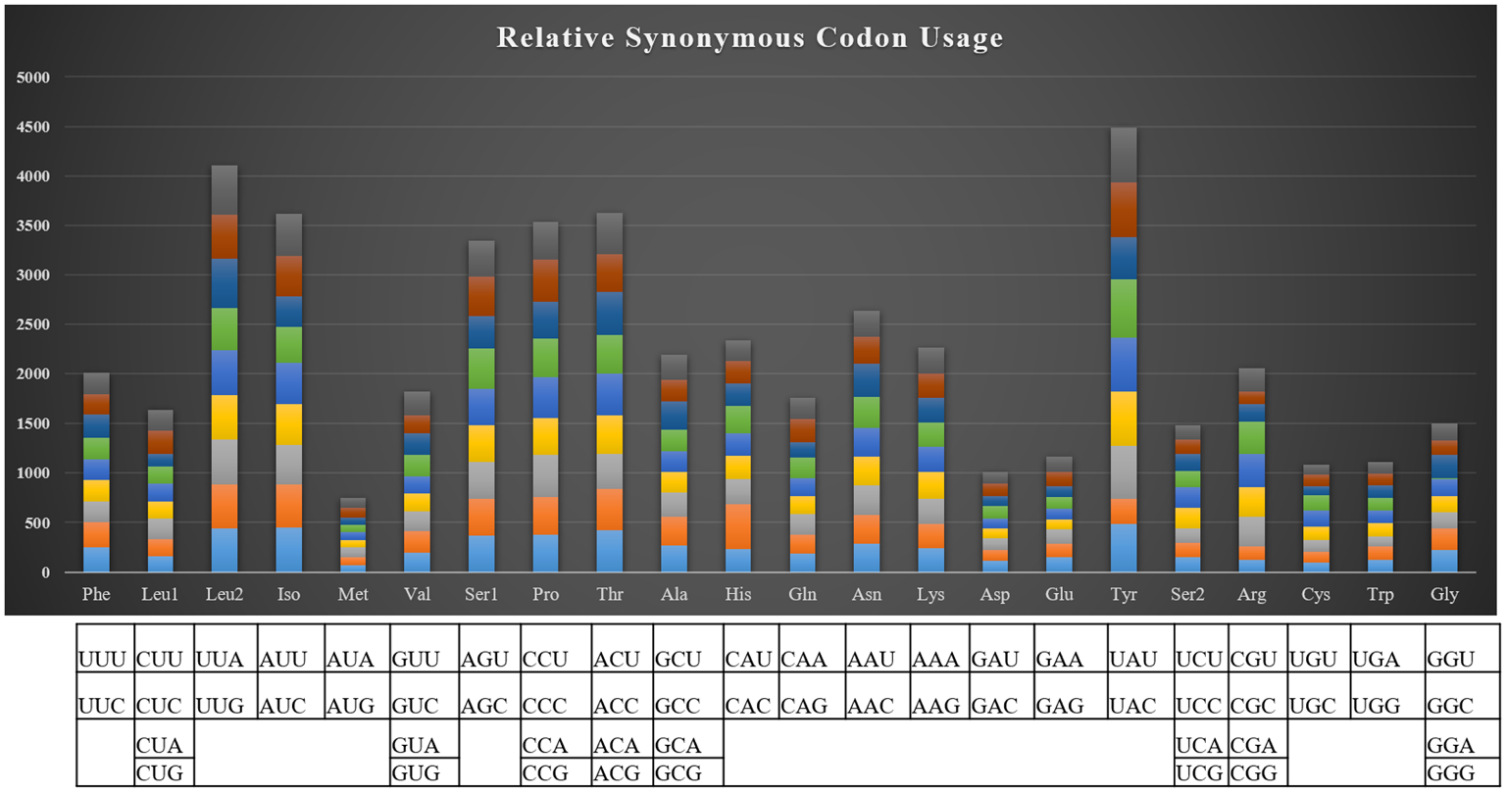
Codon usage of the mitochondrial protein coding genes of Asiatic black bear and other nine sub-species in Ursidae family (MH281753-*Ursus thibetanus laniger,* EF667005- *Ursus thibetanus ussuricus*, DQ402478-*Ursus thibetanus mupinensis*, EF587265- *Ursus thibetanus thibetanus,* EF076773-*Ursus thibetanus formosanus*, AF303109-*Ursus americanus*, MG066704-*Ursus thibetanus,* AB863014-*Ursus thibetanus japonicus*; MN935768-*Ursus thibetanus laniger (India; Present Study)*.

**Figure 5:**
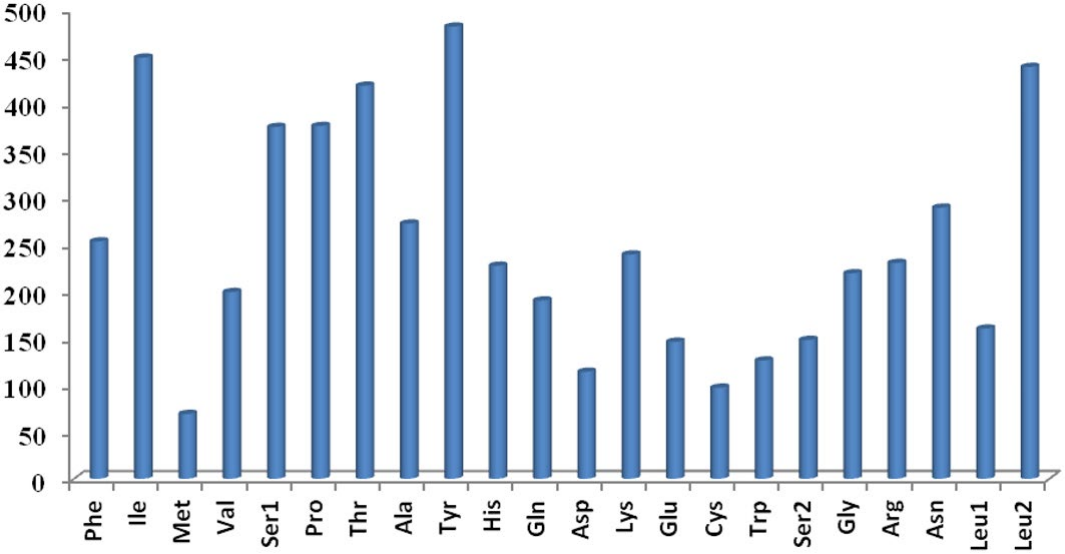
Relative synonymous codon usage (RSCU) of the mitochondrial protein-coding genes of *U. thibetanus laniger* mitochondrial genome. Codon count numbers are provided on x-axis and amino acids on y-axis.

**Figure 6:**
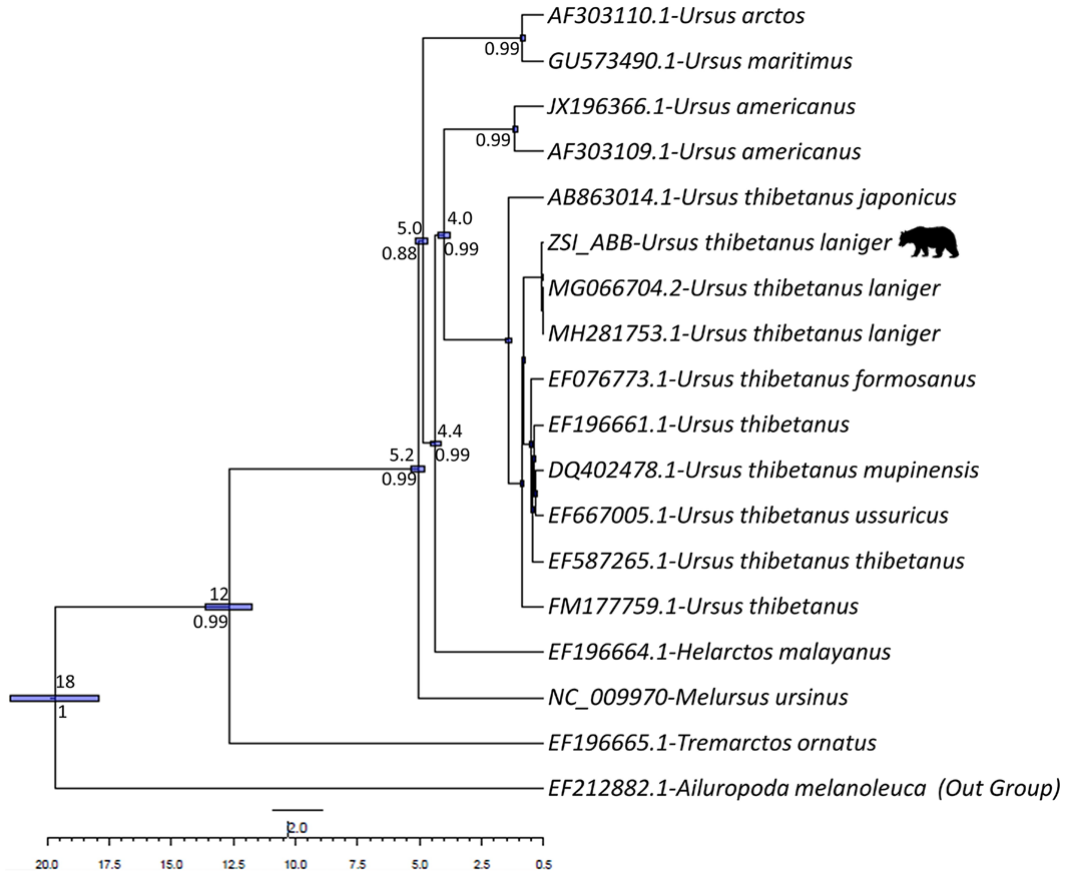
Phylogenetic relationship among Asiatic black bear and related species inferred from their whole mitochondrial genome using Bayesian inference. Posterior probability values are shown on each node. *Ailuropoda melanoleuca* (EF212882) was used as out-group.

The relative synonymous codon usage showed the highest utilization of codons of UAC, UUG, AUC and ACC among all the PCGs (Fig. 4). The RSCU analysis revealed the most occurred amino acids in protein-coding genes of *U. t. laniger* mitochondrial genome were Ile, Tyr, Leu, and Thr with 449, 482, 439 and 419 codon frequencies, respectively (Table S2, Fig. 5), whereas Met, Cys and Asp were less abundant. We did not find any difference in the RSCU of the *U. t. laniger* when compared with the other subspecies of Asiatic black bear. The phylogenetic analysis showed that the two mitogenomes i.e. MG066704.2 and MH281753.1 shared 99% similarity with the assembled mitogenome of *U.t. laniger.* These two mitogenomes were sequenced from Nepal^10^ and Tibetan Plateau-Himalaya region^27^ which are the known distribution ranges of the *U.t. laniger,* exhibiting an obvious trend of clustering in phylogeny with strong bootstrap support (Fig. 6). All mitogenomes of different sub-species of Asiatic black bear *i.e. U. t. japonicus, U. t. formosanus, U. t. ussuricus, U. t. thibetanus and U. t. mupinensis*, formed sister branches to the *U.t.laniger*. In an earlier study conducted on the sequencing of complete mitogenome of Japanese black bear, *U. t. laniger* was not included in the phylogeny due to non-availability of the sequences^28^.

Further, tRNAs secondary structure of *U .t. laniger* were compared with the other taxon of Ursids whose complete mitogenomes were available. The comparison showed more than 90% structure similarity with MH281753.1 (99%), MG066704.2 (99%), EF076773.1 (95%), EF19666.1 (93%) with z-score value of more than 10.0 and lesser similarity with EF196665.1 (39%) and EF212882.1 (46%) (Table S3) having low structure stability which was also evident from the phylogenetic analysis. We found no functional change in wobble position of anticodon (UAA) except in *Ailuropoda melanoleuca* (AAG). The pairwise genetic distances matrix, calculated based on Kimura 2-parameter model indicated that assembled mitogenome of *U. t. laniger* showed highest genetic differentiation with *U. t. mupinensis* (0.019) and lowest with the subspecies of Asiatic black bear sequenced from Nepal and Tibetan Plateau-Himalaya region (0.001), expectedly the HBB, *U. t. laniger* within the species of *U. thibetanus* (Table S4).

## Conclusion

Mitogenome analysis is imperative to make inferences on species phylogenies and resolving species divergence at different taxonomic levels^29,30^. In the present study, size of the complete mitogenome of HBB (*U.t. laniger*) was found to be 16,556 bp in length and showed similar gene order as found in other sub-species of Asiatic Black bears. Further, the tRNA secondary structure comparative analysis revealed no functional change in wobble position of anticodon except in *Ailuropoda melanoleuca.* Similar to the tRNA secondary structure, PCGs and RSCU structural comparative analysis, we did not find any change among the different subspecies of Asiatic black bear.No structural differences in tRNA, PCGs and RSCU among different subspecies of the Asiatic Black bear indicated evolutionary conserved nature of the mitochondrial genes. Bayesian tree showed distinct clusters, species wise paraphyletic clades formed, where all the six subspecies of Asiatic black bears formed sister branches and the phylogenetic relationships were congruent with the tRNA structure similarity with the other available ursids species. The findings of the present study demonstrate the detailed workflow in rapid recovery and assembly the complete mitogenome of HBB (*U.t. laniger*) from the low depth sequencing data. A close similarity (99%) of HBB from Indian Himalayan region with *U.t. laniger* from Nepal and Tibetan Plateau-Himalaya region suggested its distribution in large area and open the scope for transboundary research among range countries for population level information***. ***The study also generates opportunities to overcome the conventional strategies of obtaining long-range PCR and subsequently drawing phylogenetic inferences. The complete mitogenome reported in the present study is expected to allow for further genomics studies of the ursidae species and would be useful for conservation genetics.

## Methods

### Sample collection, DNA extraction and library preparation

We collected a small tissue portion, approx. 500 mg from a HBB carcass during the field surveys in the district of Uttarkashi of the State Uttarakhand, India. Total genomic DNA was isolated using Qiagen DNeasy Blood and Tissue Kit (Qiagen, Germany) according to the manufacturer’s instructions. The NGS was outsourced to the Xcelris Labs Pvt. Ltd. Ahmedabad, India. Approximately, 200 ng genomic DNA was sheared using Covaris S2 sonicator (Covaris, Woburn, Massachusetts, USA) to generate fragment of read length 2 × 150 bp PE. The TruSeq DNA Library Preparation kit (https://support.illumina.com/downloads/truseq) was used for the construction of the paired-end library (8 lanes) with standard protocols. The resultant library was sequenced using Illumina HiSeq 2500 (2 × 150 base paired-end reads) (Illumina, USA) platform which yielded ~ 12 million reads.

### Quality check and reference-based assembly

**Figure 7:**
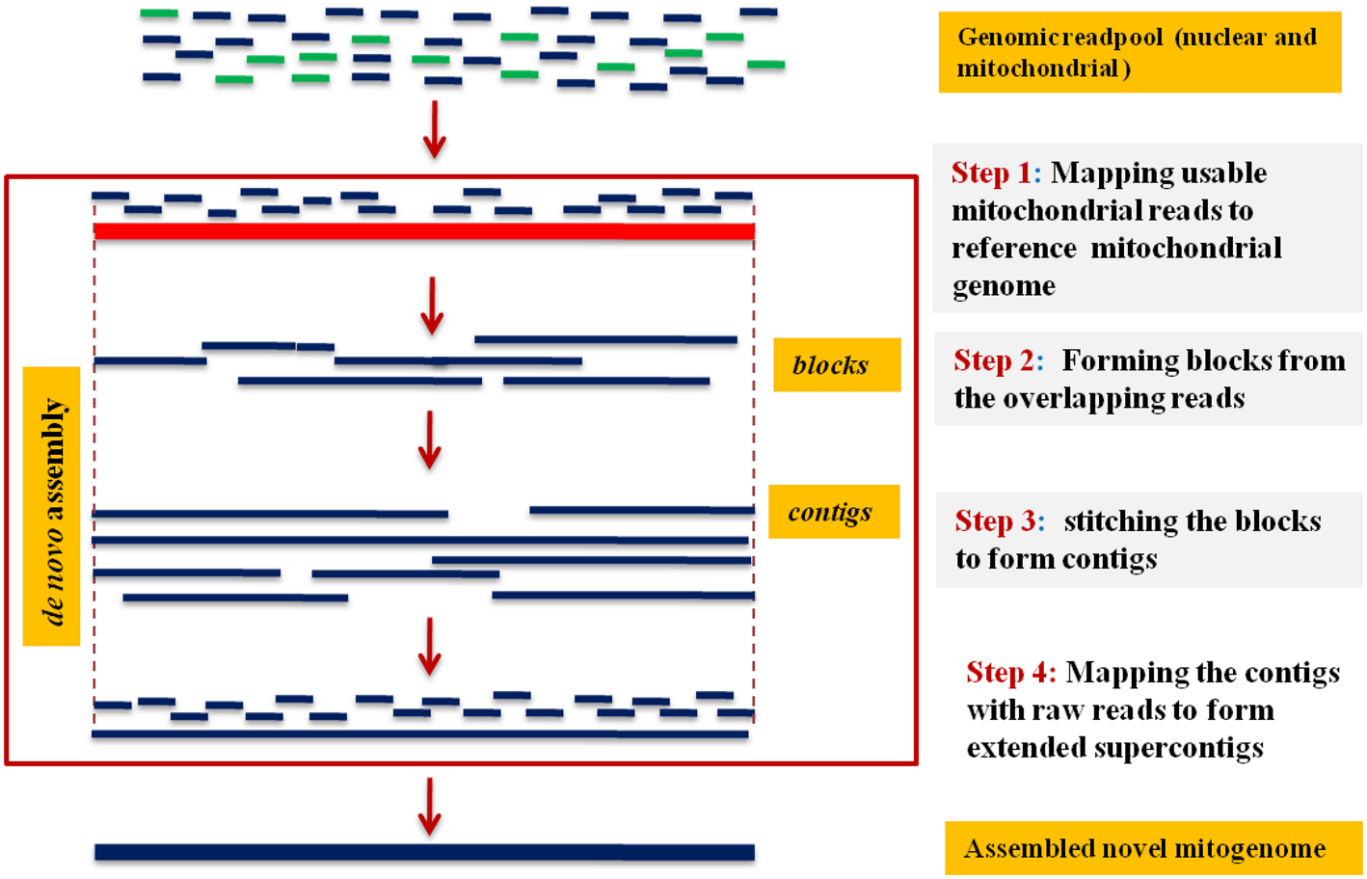
Pipeline adopted for the reference based de-novo assembly of the novel mitogenome from the genomic readpool.

Quality screening of raw reads was done using FastQC (https://www.bioinformatics.babraham.ac.uk/projects/fastqc/) and reads with low quality (Q < 20) and shorters (< 50 bp) were filtered out using NGS QC toolkit (https://www.nipgr.res.in/ngsqctoolkit.html). Usable reads were mapped against reference genome (MH281753) using bwa-aln (0.7.17) and then the fishing reads were grouped into extended reads (blocks) and the resultant contig was re-mapped with the filtered reads in order to increase the correctness of assembly using CLC genomics workbench version 12.0.3 (https://www.qiagenbioinformatics.com/products/clc-genomics-workbench/) with default parameters One of the longest contigs that represented the assembled complete mitochondrial genome of HBB, was thus generated (Fig. 7).

### Genome characterization and comparative analysis

The circular representation of the generated mitogenome was viewed using CGView Server (https://stothard.afns.ualberta.ca/cgview_server/)^31^. The 22 tRNA genes were verified using tRNAscan-SE software^32^ using the mammalian mitochondrial genetic code under the default mode. The gene arrangement and their order were verified using MITOS online server (https://mitos.bioinf.uni-leipzig.de). The overlapping regions and intergenic spacers were counted manually using Microsoft Excel (2007). The start and stop codon of Protein Coding Genes (PCGs) were checked through Open Reading Frame Finder (https://www.ncbi.nlm.nih.gov/orffinder/) web tool. The base skewness was calculated using AT skew = [A − T]/[A + T], GC skew = [G − C]/[G + C]^33^ and Short Sequence repeats (SSRs) were screened using MISA-web software^34^ and tandem repeats were identified using Tandem Repeat Finder (https://tandem.bu.edu/trf/trf.html) web tool^35^. AT-GC content and its skewness of mitogenome *U. t. laniger* sequences were compared with other sub-species of black bear. The comparative analysis of Relative Synonymous Codon Usage (RSCU) and codon distribution of *U. t. laniger* sequences and other sub-species of black bear were calculated using MEGA X^36^. Comparative analysis in the consistency of the secondary structure of tRNA of *U. thibetanus* with other available complete mitogenome of Ursidae family was done using web-beagle (https://beagle.bio.uniroma2.it/) aligner^37^.

### Genetic distance and phylogenetic re-construction

We also downloaded 17 mitogenomes, *i.e.* one each from the six subspecies of Asiatic black bear -*U.t.ussuricus, U.t. mupinensis*, *U.t. thibetanus, U.t. formosanus, U.t.laniger, U.t.japonicus,* two mitogenomes of American black bear (*Ursus americanus*), one each from brown bear (*Ursus arctos*), polar bear (*Ursus maritimus*), sun bear (*Helarctos malayanus*), Sloth bear (*Melursus ursinus*), Spectable bear (*Tremarctos ornatus*)*,* Giant panda (*Ailuropoda melanoleuca*)*,* and three mitogenomes of unassigned subspecies of Asiatic black bear (Table 3). The pair-wise genetic distances were estimated using MEGA X^36^ among the different subspecies of Asiatic black bear and the other taxon of Ursids. Bayesian-based phylogeny among the mentioned taxon of Ursids was reconstructed using BEAUti v 1.6.1 and BEAST v.1.10.4^38^ considering *Ailuropoda melanoleuca* as an out-group. We applied the best fit model HKY selected by Model test 3.6^39^ with BIC criteria. For the molecular clock rate, we used a normal prior divergence as used by Wayne et al.^40^ by placing a standard deviation on the rate equals to 10% of the mean to account for variation and uncertainty in the rate. Dating analyses were performed for 20 million generations while sampling every 1000th tree, and the first 10% of trees sampled were treated as burn-in, and visualized in Figtree v 1.4.4^41^.

## Supplementary information

Supplementary Information 1.
