# Supplementary material for: Assembling mitogenome of Himalayan Black Bear (U. t. laniger) from low depth reads and its application in drawing phylogenetic inferences
Source: Sci Rep. 2021 Jan 12;11:730. doi: 10.1038/s41598-020-76872-y (PMC7803731; doi:10.1038/s41598-020-76872-y)
Supplement: Supplementary file 1 — Supplementary Information 1. [file 41598_2020_76872_MOESM1_ESM.doc]

**Assembling mitogenome of Himalayan Black Bear (*U. t. laniger*) from low depth reads and its application in drawing phylogenetic inferences**

**Amrita Bit, MukeshThakur, Sujeet Kumar Singh, Bheem Dutt Joshi , Vinay Kumar Singh, Lalit Kumar Sharma, Basudev Tripathy, Kailash Chandra**

**Table S1:** Identifictaion of the repeat region (SSRs/STRs)

| **SSR/ STR** | **SSR** | **Size** | **start** | **end** |
| --- | --- | --- | --- | --- |
| SSR | (AT)4 | 8 | 15842 | 15849 |
| STR | (TGTACGCATA)7 | 70 | 16485 | 16554 |

**Table S2: Codon number and Relative synonymous codon usage in Himalayan black bear, *Ursus thibetanus laniger*** mitochondrial genome.

| **Codon** | **Count** | **RSCU** | **Codon** | **Count** | **RSCU** | **Codon** | **Count** | **RSCU** | **Codon** | **Count** | **RSCU** |
| --- | --- | --- | --- | --- | --- | --- | --- | --- | --- | --- | --- |
| UUU(F) | 123 | 0.97 | UCU(S) | 110 | 1.26 | UAU(Y) | 145 | 1.14 | UGU(C) | 49 | 1.01 |
| UUC(F) | 130 | 1.03 | UCC(S) | 108 | 1.24 | UAC(Y) | 110 | 0.86 | UGC(C) | 48 | 0.99 |
| UUA(L) | 115 | 1.15 | UCA(S) | 123 | 1.41 | UAA(Y) | 140 | 1.32 | UGA(W) | 92 | 0.87 |
| UUG(L) | 45 | 0.45 | UCG(S) | 34 | 0.39 | UAG(Y) | 87 | 0.82 | UGG(W) | 34 | 1 |
| CUU(L) | 122 | 1.22 | CCU(P) | 116 | 1.23 | CAU(H) | 129 | 1.14 | CGU(R) | 28 | 0.73 |
| CUC(L) | 90 | 0.9 | CCC(P) | 115 | 1.22 | CAC(H) | 98 | 0.86 | CGC(R) | 32 | 0.83 |
| CUA(L) | 169 | 1.69 | CCA(P) | 115 | 1.22 | CAA(Q) | 134 | 1.41 | CGA(R) | 40 | 1.04 |
| CUG(L) | 58 | 0.58 | CCG(P) | 30 | 0.32 | CAG(Q) | 56 | 0.59 | CGG(R) | 26 | 0.68 |
| AUU(I) | 147 | 0.98 | ACU(T) | 147 | 1.4 | AAU(N) | 168 | 1.16 | AGU(S) | 64 | 0.73 |
| AUC(I) | 126 | 0.84 | ACC(T) | 108 | 1.03 | AAC(N) | 121 | 0.84 | AGC(S) | 84 | 0.96 |
| AUA(I) | 176 | 1.18 | ACA(T) | 122 | 1.16 | AAA(K) | 164 | 1.37 | AGA(R) | 60 | 1.57 |
| AUG(M) | 69 | 1 | ACG(T) | 42 | 0.4 | AAG(K) | 75 | 0.63 | AGG(R) | 44 | 1.15 |
| GUU(V) | 45 | 0.9 | GCU(A) | 72 | 1.06 | GAU(D) | 63 | 1.11 | GGU(G) | 41 | 0.75 |
| GUC(V) | 50 | 1.01 | GCC(A) | 89 | 1.31 | GAC(D) | 51 | 0.89 | GGC(G) | 53 | 0.97 |
| GUA(V) | 75 | 1.51 | GCA(A) | 90 | 1.32 | GAA(E) | 90 | 1.23 | GGA(G) | 79 | 1.44 |
| GUG(V) | 29 | 0.58 | GCG(A) | 21 | 0.31 | GAG(E) | 56 | 0.77 | GGG(G) | 46 | 0.84 |

**Table S3: tRNA structure similarity comparison of Himalayan black bear, *Ursus thibetanus laniger*** **with other available species in Ursidae.**

| **RNA1 (accession no.)** | **RNA2 (accession no.)** | **Seq ID(%)** | **Str ID (%)** | **P-value** | **Z-score** | **Anticodon** |
| --- | --- | --- | --- | --- | --- | --- |
| MN935768 | EF196665 | 82.03 | 22.02 | 0.0001 | 3.056 | UAA |
| MN935768 | EF212882 | 86.74 | 27.79 | 0.0001 | 4.185 | AAG |
| MN935768 | JX196366 | 95.56 | 29.91 | 0.0001 | 4.797 | UAA |
| MN935768 | NC009970 | 94.96 | 34.42 | 0.0001 | 5.541 | UAA |
| MN935768 | GU573490 | 96.49 | 34.55 | 0.0001 | 5.36 | UAA |
| MN935768 | EF196664 | 92.64 | 40.25 | 0.0001 | 5.644 | UAA |
| MN935768 | AF303110 | 90.12 | 43.44 | 0.0001 | 5.875 | UAA |
| MN935768 | AB863014 | 96.95 | 47.94 | 0.0001 | 6.579 | UAA |
| MN935768 | AF303109 | 93.77 | 51.59 | 0.0001 | 6.93 | UAA |
| MN935768 | FM177759 | 96.49 | 67.44 | 0.0001 | 8.535 | UAA |
| MN935768 | DQ402478 | 98.14 | 69.1 | 0.0001 | 9.186 | UAA |
| MN935768 | E667005 | 95.16 | 77.19 | 0.0001 | 9.242 | UAA |
| MN935768 | EF587265 | 99.01 | 82.69 | 0.0001 | 10.39 | UAA |
| MN935768 | EF196661 | 98.94 | 90.92 | 0.0001 | 10.839 | UAA |
| MN935768 | EF076773 | 99.2 | 92.31 | 0.0001 | 11.085 | UAA |
| MN935768 | MH281753 | 99.93 | 98.87 | 0.0001 | 11.691 | UAA |
| MN935768 | MG066704 | 99.93 | 98.87 | 0.0001 | 11.691 | UAA |

**Table S4: Genetic distance among the sub-species and closely related species.**

|  | 1 | 2 | 3 | 4 | 5 | 6 | 7 | 8 | 9 | 10 | 11 | 12 | 13 | 14 | 15 | 16 | 17 |
| --- | --- | --- | --- | --- | --- | --- | --- | --- | --- | --- | --- | --- | --- | --- | --- | --- | --- |
| MN935768 (1) | 0.000 |  |  |  |  |  |  |  |  |  |  |  |  |  |  |  |  |
| MH281753.1 (2) | 0.001 |  |  |  |  |  |  |  |  |  |  |  |  |  |  |  |  |
| MG066704.2 (3) | 0.001 | 0 |  |  |  |  |  |  |  |  |  |  |  |  |  |  |  |
| EF196661.1 (4) | 0.01 | 0.011 | 0.011 |  |  |  |  |  |  |  |  |  |  |  |  |  |  |
| EF667005.1 (5) | 0.012 | 0.013 | 0.013 | 0.005 |  |  |  |  |  |  |  |  |  |  |  |  |  |
| FM177759.1 (6) | 0.011 | 0.011 | 0.011 | 0.011 | 0.015 |  |  |  |  |  |  |  |  |  |  |  |  |
| DQ402478.1 (7) | 0.019 | 0.02 | 0.02 | 0.015 | 0.015 | 0.022 |  |  |  |  |  |  |  |  |  |  |  |
| EF587265.1 (8) | 0.009 | 0.009 | 0.009 | 0.005 | 0.007 | 0.011 | 0.015 |  |  |  |  |  |  |  |  |  |  |
| EF076773.1 (9) | 0.007 | 0.008 | 0.008 | 0.007 | 0.009 | 0.011 | 0.017 | 0.005 |  |  |  |  |  |  |  |  |  |
| AB863014.1 (10) | 0.015 | 0.015 | 0.015 | 0.017 | 0.019 | 0.018 | 0.023 | 0.015 | 0.015 |  |  |  |  |  |  |  |  |
| JX196366.1 (11) | 0.036 | 0.036 | 0.036 | 0.034 | 0.035 | 0.034 | 0.039 | 0.033 | 0.035 | 0.036 |  |  |  |  |  |  |  |
| AF303109.1 (12) | 0.033 | 0.034 | 0.034 | 0.032 | 0.031 | 0.033 | 0.034 | 0.03 | 0.032 | 0.033 | 0.015 |  |  |  |  |  |  |
| AF303110.1 (13) | 0.039 | 0.04 | 0.04 | 0.037 | 0.039 | 0.039 | 0.039 | 0.037 | 0.04 | 0.041 | 0.043 | 0.04 |  |  |  |  |  |
| NC009970 (14) | 0.038 | 0.039 | 0.039 | 0.035 | 0.039 | 0.039 | 0.042 | 0.033 | 0.036 | 0.039 | 0.044 | 0.04 | 0.047 |  |  |  |  |
| GU573490.1 (15) | 0.04 | 0.041 | 0.041 | 0.036 | 0.039 | 0.041 | 0.04 | 0.036 | 0.039 | 0.044 | 0.047 | 0.042 | 0.013 | 0.046 |  |  |  |
| EF196665.1 (16) | 0.083 | 0.084 | 0.084 | 0.083 | 0.084 | 0.085 | 0.092 | 0.084 | 0.087 | 0.084 | 0.081 | 0.083 | 0.075 | 0.087 | 0.079 |  |  |
| EF196664.1 (17) | 0.038 | 0.039 | 0.039 | 0.037 | 0.037 | 0.041 | 0.041 | 0.039 | 0.04 | 0.038 | 0.043 | 0.039 | 0.043 | 0.047 | 0.049 | 0.083 | 0 |

*Number given in the above diagnol of table is represtented with their accesiion numbers under first column of table.*
